# LPA5 Is Abundantly Expressed by Human Mast Cells and Important for Lysophosphatidic Acid Induced MIP-1β Release

**DOI:** 10.1371/journal.pone.0018192

**Published:** 2011-03-28

**Authors:** Anders Lundequist, Joshua A. Boyce

**Affiliations:** 1 Department of Medicine, Harvard Medical School, Boston, Massachusetts, United States of America; 2 Division of Rheumatology, Immunology, and Allergy, Brigham and Women's Hospital, Boston, Massachusetts, United States of America; 3 Anatomy, Physiology & Biochemistry, Swedish University of Agricultural Sciences, Uppsala, Sweden; University of Muenster, Germany

## Abstract

**Background:**

Lysophosphatidic acid (LPA) is a bioactive lipid inducing proliferation, differentiation as well as cytokine release by mast cells through G-protein coupled receptors. Recently GPR92/LPA5 was identified as an LPA receptor highly expressed by cells of the immune system, which prompted us to investigate its presence and influence on mast cells.

**Principal Findings:**

Transcript analysis using quantitative real-time PCR revealed that LPA5 is the most prevalent LPA-receptor in human mast cells. Reduction of LPA5 levels using shRNA reduced calcium flux and abolished MIP-1β release in response to LPA.

**Conclusions:**

LPA5 is a bona fide LPA receptor on human mast cells responsible for the majority of LPA induced MIP-1β release.

## Introduction

Mast cells (MCs) are important effector cells in allergy, innate and adaptive immunity, cardiovascular disease, and cancer (reviewed in [1]). Their strategic localization around the microvasculature, in the skin, and in the respiratory and gastrointestinal tracts situates them ideally to be first responders to infectious agents and inflammatory insults. Activated MCs release chemoattractants and mediators affecting the vascular permeability, leading to plasma extravasation and facilitating emigration of circulating inflammatory cells to the site of MC activation. In allergic diseases, the most important mechanism responsible for MC activation is cross-linking of the high affinity IgE-receptor FceRI by allergens. MC-dependent innate immune responses reflect their activation through several receptors for bacterial products (e.g. toll-like receptor (TLR)-2, TLR-4) and for complement fragments (e.g., C5a) [2], [3]. The factors that contribute to MC activation in cardiovascular disease and cancer are unknown.

Lysophosphatidic acid (LPA) is a prominent lipid component of serum that has pleiotropic effects in cell development, inflammation, and cancer [4], [5], [6]. The biosynthesis of LPA involves either the sequential action of secretory phospholipase A (sPLA) and lysophospholipase D or phospholipase D followed by cytosolic PLA, and can also be achieved by phosphorylation of monoacylglycerol (MAG) by MAG kinases [7]. The most prominent pathway responsible for maintaining extracellular LPA levels in the circulation stems from the action of the lysoPLD known as autotaxin, an enzyme intimately associated with the progression of tumors and metastasis [8]. LPA is present in the serum at micromolar concentrations, circulating bound to serum proteins, particularly to albumin and gelsolin. In response to inflammation, LPA diffuses into the extravascular space, where it exerts a wide variety of biological activities, including chemotaxis, proliferation, survival, cytokine and chemokine secretion, platelet aggregation and smooth muscle cell contraction [9], [10], [11].

Many of the effects of LPA are mediated by specific cell surface receptors. To date, eight G-protein coupled receptors (GPCRs) that recognize LPA have been reported, including three members of the endothelial differentiation and growth (Edg)-family (LPA1, LPA2, and LPA3), and five of the purinergic (P2Y)-like family (LPA4 (GPR23), LPA5 (GPR92), LPA6 (also known as P2Y5), P2Y10 and GPR87 [12], [13], [14], [15], [16], [17], [18], [19]). We previously reported that human MCs (hMCs) derived from cord blood express LPA1, LPA2, LPA3, and LPA4 mRNA. hMCs proliferate and generate cytokines in response to LPA, suggesting that LPA may be an activating ligand for MCs in circumstances where it is abundant in the extravascular space, such as cancer or myocardial infarction [4]. Based on experiments using receptor-selective agonists and antagonists, LPA-induced proliferation of hMCs was attributed to the functions of LPA1 and LPA3, whereas cytokine generation reflected LPA2 [20]. Subsequent to those studies, the orphan receptor GPR92 was found to bind LPA and was re-designated as LPA5, and found to be particularly highly expressed on cells associated with the immune system [21].

In the present study, we demonstrate that LPA5 is the most abundant LPA receptor expressed by human MCs at the mRNA level. Using short hairpin RNA knockdown, we demonstrate that LPA5 is involved in LPA-induced calcium flux, particularly at low ligand concentrations, and crucial for the generation of the chemokine macrophage inflammatory protein-1β (MIP-1β) response to LPA. MIP-1β is a potent chemoattractant for, and activator of, monocytes, lymphocytes and a wide variety of immune cells [22]. We conclude that LPA5 may mediate a pathway for MC activation in circumstances where cancers or cardiovascular inflammation result in the local production of LPA.

## Materials and Methods

### Reagents

RT^2^ FAST SYBR Green (PA-042), RT^2^ First strand kit (C-03) and primers were from SABiosciences (LPA1; PPH02360A, LPA2; PPH02343A, LPA3; PPH02354A, LPA4; PPH01859A, LPA5; PPH12977A, P2Y10; PPH16348B, GPR87; PPH09602A, GAPDH; PPH00150E). Antibodies directed against either an extracellular epitope (LS-A425) or an intracellular epitope (LS-A426) were from MBL International, whereas FITC-labeled goat anti-rabbit IgG (554020) was from BD Biosciences.

TRI Reagent (T9424) was from Sigma-Aldrich.

### Cell culture

The LAD2 line [23] isolated from the bone marrow of a patient with MC leukemia was a gift from A. Kirshenbaum (National Institutes of Health). These cells were cultured in StemPro-34 (Invitrogen, 10639-011) supplemented with 2 mM L-glutamine (Invitrogen), 100 IU/ml Pen-strep (Invitrogen), and 100 ng/ml stem cell factor (SCF; Thermo Fisher Scientific). Cell culture medium was hemidepleted every week with fresh medium and 100 ng/ml SCF. Primary human cord blood MCs (hMCs) were derived from cord blood mononuclear cells using SCF (100 ng/ml), IL-6 (Peprotech, 200-06, 50 ng/ml), and IL-10 (Peprotech, 200-10, 10 ng/ml) as described [24] and used when >95% stained with toluidine blue (typically 6 wk).

### Cell activation

LAD2 cells were stimulated with the indicated concentrations of LPA (D (+)-sn-1-O-oleoyl-glyceryl-3-phosphate, Echelon Bioscience, UT, US) or were passively sensitized with 2 µg/ml of human myeloma IgE (Millipore, AG30P-K) overnight and stimulated with 1 µg/ml of goat anti–human anti-IgE (Millipore, AP175) as detailed elsewhere [25]; unstimulated LAD2 cells were used as controls. The concentration of secreted MIP-1β in conditioned media from LAD2 cells was measured by an ELISA (Invitrogen CHC2293).

### Calcium flux

Cells were washed once in cold HBSS, 1 mM CaCl_2_, 1 mM MgCl_2_ (CM buffer) and subsequently resuspended at 1×10^6^ ells/ml in CM buffer containing 2.5 µg/ml Fura 2-AM (Molecular Probes) followed by incubation for 30 min at 37°C. Excess Fura 2-AM was removed by washing twice with CM buffer, and the cells were finally resuspended in CM buffer at 0.5×10^6^ cells/ml. Cells were stimulated with the indicated concentrations of LPA, and changes in intracellular calcium concentration were measured using excitation at 340 and 380 nm in a fluorescence spectrophotometer (F-4500; Hitachi). The relative ratios of fluorescence emitted at 510 nm were recorded and displayed as a reflection of intracellular calcium concentration.

### Targeted suppression of LPA5 transcript

shRNA constructs were purchased from Open Biosystems (Huntsville, AL). The constructs were designed to include a hairpin of 21 base pair of a sense strand and an antisense strand, and a 6 base-pair loop. The sequence of the most efficient construct TRCN0000008981 was as follows:

5′-CCGGGCTGTGCTTCGTGCCCTACAACTCGAGTTGTAGGGCACGAAG CACAGCTTTTT-3′

Infectious viral particles were prepared by co-transfection of 293FT cells with an shRNA expressing plasmid, or a control plasmid, and a lentiviral packaging mix (Virapower K4975-00; Invitrogen) according to the manufacturer's protocol. The viral stocks were titered in HeLa cells and added to LAD2 cells (0.25×10^6^ cell/ml; 1 ml, 24 well plate) at an MOI of 10. The cells were incubated overnight with the particles at 37°C. The following day, the medium was replaced and the cells were cultured for an additional 48 hours before performing functional studies.

### Expression analysis

Total RNA was extracted from cells using TRI Reagent and further purified using Qiagen RNEasy where a DNAse step was included to eliminate genomic DNA contaminations. The RNA concentration was determined by absorbance readings at 260 nm, whereas the ratio of absorbance at 260/280 nm and 260/230 nm was used to assess purity. 100 ng of samples with 260/280 readings of >1.8 and 260/230 readings of >1.9 were subsequently converted into cDNA using RT^2^ First strand kit according to the manufacturer's instruction. Transcript levels were determined using a SYBR Green based system containing ROX as passive reference and run on a Stratagene Mx3000p machine (95°C, 10 min; 40 cycles of 95°C, 15 sec; 60°C, 60 sec). The mRNA expression was normalized to the expression of GAPDH in individual samples.

### Flow cytometry

Surface expression of LPA5 on LAD2 and hMCs was assessed by staining non-permeabilized cells with a polyclonal antibody (MBL International, LS-A425, diluted 25 times) followed by incubation with a FITC-labeled goat anti-rabbit IgG (BD Biosciences, 554020, diluted 200 times). The cells were detected on a FACSCanto flow cytometer (BD Biosciences). Analysis was conducted with FlowJo 7.2.

Statistical analysis was performed in GraphPad Prism 4, using Two-sided Student's t-test.

## Results and Discussion

### Expression analysis of LPA receptors on MCs

**Figure 1:**
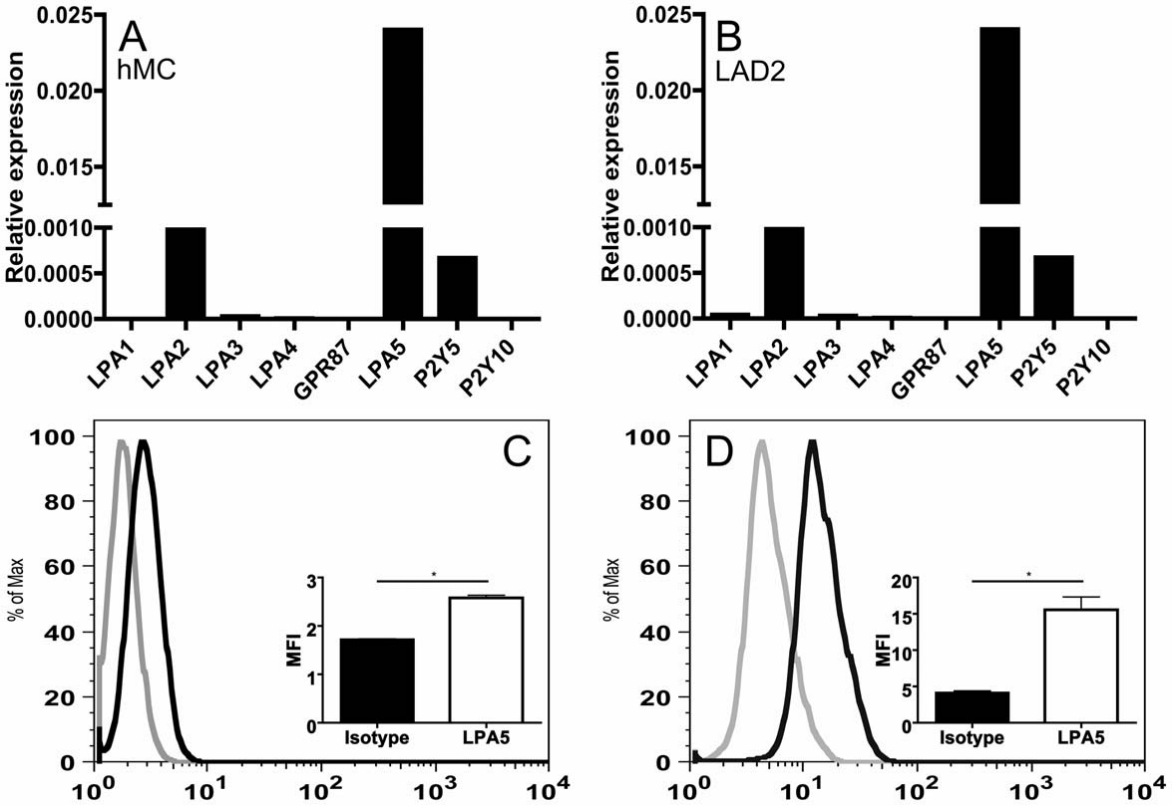
Expression of LPARs by LAD2 cells and hMCs. The expression of LPARs in hMC (A) and LAD2 (B) was assessed using SYBR green based quantitative real-time PCR; transcript levels are normalized to the expression of GAPDH in individual samples (representative data of 3 determinations). Flow cytometric analysis was used to verify presence of LPA5 on the surface of unpermeabilized hMC (C) and LAD2 (D); grey tracing represents isotype control whereas black tracing represents specific LPA5 staining. Mean fluorescence difference compared to isotype depicted in insets of respective cell type (Mean MFI ± SD, N = 3 independent experiments, *P<0.05).

The recent identification of LPA5 as being able to interact with LPA prompted us to investigate its expression in human MCs. Quantitative real-time PCR (qPCR) analysis revealed that mRNA encoding LPA5 was the most abundant LPA receptor transcript in both primary hMCs (Fig. 1a) and the well-differentiated MC sarcoma line, LAD2 by several fold over LPA1, LPA2, LPA3, LPA4, GPR87, P2Y5 and P2Y10) (Fig. 1b). Albeit less differentiated compared to fully mature MCs, LAD2 cells and primary hMCs serve as valid model systems for investigation of MC [26]. To determine whether the LPA5 transcript was translated into measurable amounts of protein, we used a polyclonal antibody against this receptor in both flow cytometry and western blot. We confirmed that LPA5 protein was readily detected on the surface of both primary hMCs (Fig. 1c) and LAD2 cells (Fig. 1d). Primary hMC display fewer LPA5 receptors despite comparable amount of transcript, an effect possibly due to differences in culturing conditions, degree of maturation or differences in regulation of translation. The observed differences in expression could influence the relative ability of each cell type to respond to LPA. Western blotting with the same antibody revealed a single band of 42 kDa, consistent with the predicted size of the LPA5 protein (not shown).

### Contribution of LPA5 to LPA-induced signaling responses in LAD2 cells

**Figure 2:**
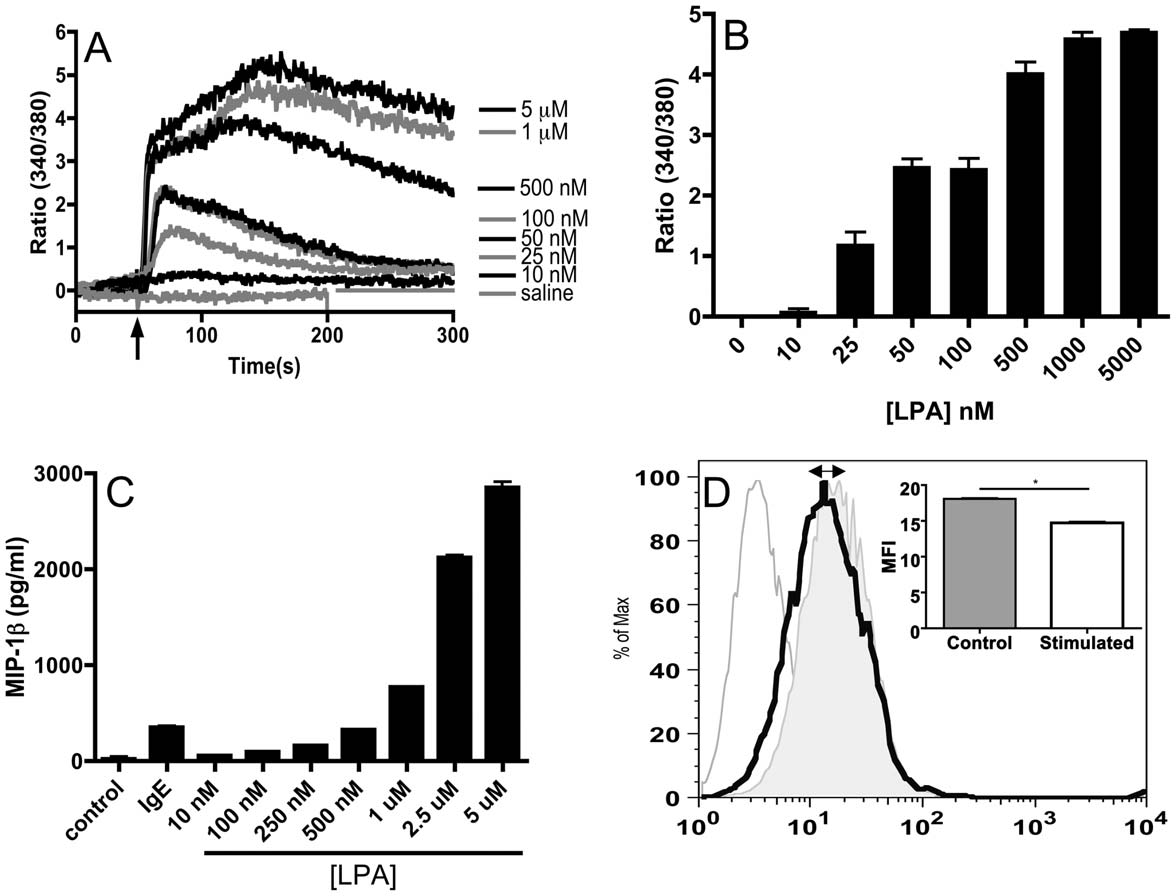
Functional responses in LAD2 induced by LPA. (A) Changes in intracellular calcium levels were determined using FURA-2AM loaded LAD2 cells (1×10^6^ cell/sample) exposed to a range of concentrations of LPA (N = 2 independent experiments). (B) Maximal calcium flux in (A) represented in bar graph form; N = 2, mean± SEM. (C) Dose-dependent release of MIP-1β from LAD2 stimulated for 6 hours with IgE-crosslinking or the indicated concentrations of LPA (N = 3 independent experiments). Mean ± SD. (D) LPA5 internalization in response to 1 mM LPA measured 15 min post stimulation determined by flow cytometric analysis. Filled grey tracing represents unperturbed cells whereas black tracing depicts LPA-stimulated cells (representative of three experiments). The quantified reduction in MFI is shown in inset (Mean ± SD, N = 5, *P<0.0001).

We had previously inferred functions of LPA1, LPA2, and LPA3 receptors in LPA-induced activation and proliferation of primary hMCs. Because of the growing number of known LPAR receptors, the ability to discriminate functions of individual receptors can be complicated by potential off-target effects of inhibitors and agonists. We therefore used an RNA interference (RNAi) approach to elucidate what biological influence LPA5 may exert on MCs. Because LAD2 cells expressed LPA5 receptors at levels comparable to that of primary hMCs and are easy to maintain and transfect, we focused our analysis on this cell line. LAD2 cells responded to LPA with a dose-dependent calcium flux, an effect associated with subsequent chemokine generation (Fig. 2a&b) [27]. Hence, we investigated whether LPA could induce MIP-1β generation, which is a hallmark chemokine associated with mast cell activation [28]. Exposing LAD2 cells to increasing doses of LPA caused a dose-dependent release of MIP-1β (Fig. 2c).Interaction between a ligand and its GPCR often causes internalization of the receptor, and treatment of LAD2 cells with 1 mM LPA for 15 minutes lead to a reduction of the levels of LPA5 receptor at the cell surface (MFI = 18.08±0.13, N = 4 vs. MFI = 14.72±0.19, N = 5, p<0.0001), suggesting some degree of ligand-induced internalization (Fig. 2d). In contrast to murine MCs [29], LAD2 cells did not degranulate in response to LPA as determined by β-hexosaminidase release (data not shown).

At low concentrations (<0.5 µM), LPA induced a transient increase in intracellular calcium levels in LAD2 cells, whereas higher concentrations of LPA caused a sustained elevation of calcium concentration. The concentration of LPA needed to induce sustained calcium levels coincides with the dose of LPA required for MIP-1β release, suggesting that an intracellular calcium concentration threshold is involved in LPA-induced generation of this chemokine.

**Figure 3:**
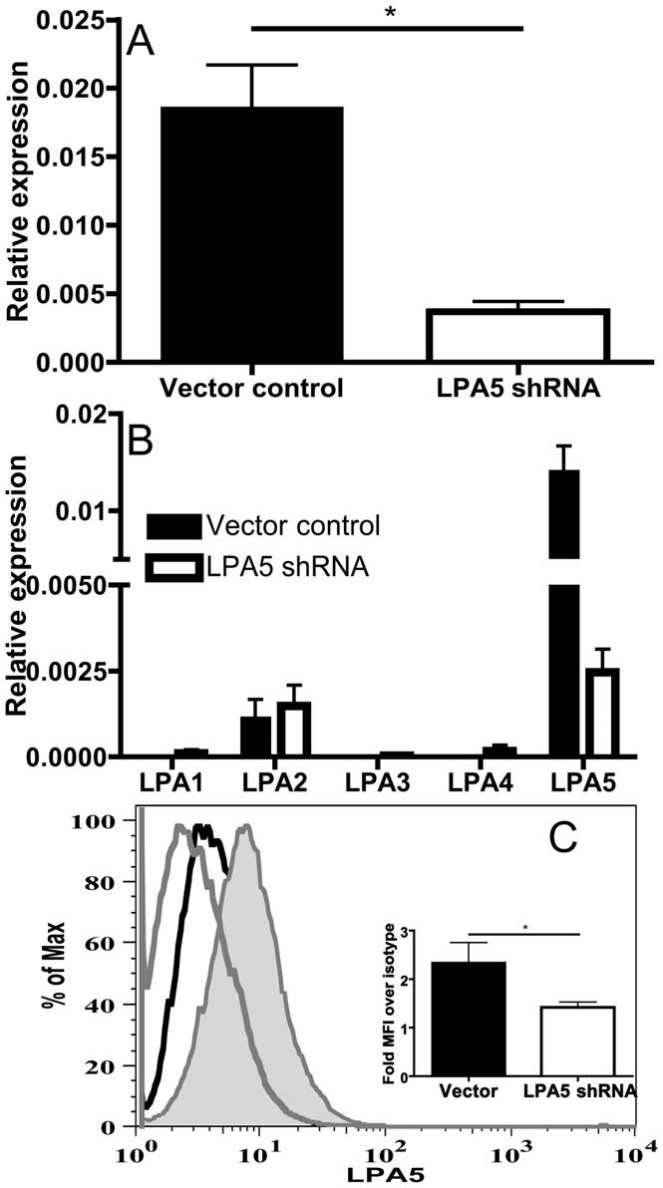
Impact of shRNA based silencing on LPA5 transcript and surface expression. (A) Transcript levels of LPA5 were determined 72 h post transduction with either control vector or LPA5 shRNA in four independent experiments. (Mean ± SD, N = 7, *P<0.05) (B) Transcript levels of a panel of LPARs were assessed to investigate possible off-target effects of transduction with vector (black bars) or LPA5 shRNA (white bars). (C) Surface expression of LPA5 after transduction with empty vector (black tracing) or LPA5 shRNA (filled, black tracing) compared to isotype control (grey tracing). The quantified reduction in MFI, determined in three independent experiments, is shown in inset (Mean ± SD, N = 5, *P<0.05).

**Figure 4:**
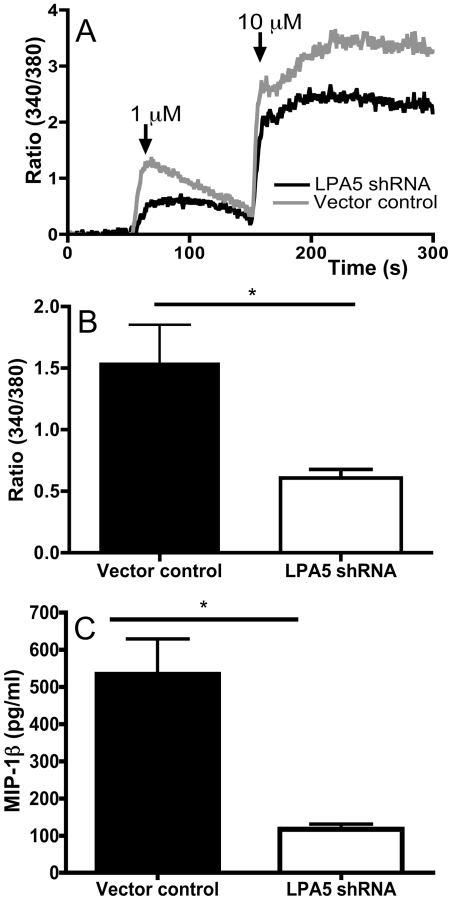
Influence of LPA5 silencing on functional responses in LAD2 cells. (A) Calcium flux in response to the indicated concentrations of LPA in control (grey tracing) or shRNA treated cells (black tracing). (B) Maximum calcium flux in response to 1 µM LPA represented in bar graph form (N = 3 Mean ± SD, *P<0.05). (C) MIP-1β release six hours after exposure to 1 µM LPA from cells transduced with control vector or LPA5 shRNA (N = 3 Mean ± SD, *P<0.05). Determinations performed in three independent experiments.

To establish the role of LPA5 in mediating calcium responses and cytokine generation in LAD2 cells, we transfected the LAD2 cells with a lentivirus encoding short hairpin (sh)RNA specifically targeting the LPA5 transcript. This maneuver reduced LPA5 mRNA levels from a mean relative expression of 0.018 (vector control) to 0.0037 (LPA5 shRNA) (Fig. 3a) with little influence on other members of the LPAR family as determined by quantitative real-time PCR (Fig. 3b). The reduction in transcript level was associated with a similar result on protein level as shown by the decrease in surface staining represented as fold difference over isotype (Fold increase of MFI over isotype  =  2.34±0.41 N = 3 vs. 1.42±0.11 N = 5, p = 0.034) (Fig. 3c). Compared with LAD2 cells treated with vector control, LPA5 shRNA-transfected LAD2 cells showed slightly reduced calcium flux in response to LPA (1.54±0.31 vs. 0.61±0.07 in response to 1 µM LPA, (Fig. 4a&b), and demonstrated markedly reduced generation of MIP-1β (535±94 pg/ml vs 117±14 pg/ml) that paralleled the reduction in LPA5 expression (Fig. 4c).

Our findings indicated that LPA5 is the dominant LPA receptor in hMCs under naïve conditions. In a previous study using pharmacologic agonists, we had implicated LPA2 receptors in LPA-mediated chemokine generation by primary hMCs. This study predated the identification of LPA5 as an LPA receptor. It is very likely that additional LPA receptors (such as LPA2) account for the residual LPA-induced calcium flux observed in LAD2 cells subjected to the knockdown of LPA5 (which resulted in a >80% depletion of transcript and significant reduction in protein, Fig. 3). Nonetheless, our data suggest that LPA5 is essential for LPA-induced signaling events that result in MIP-1β generation. It seems likely that LPA5 signals synergistically with other LPA receptors on MCs, such as LPA2. The current study provides strong evidence towards LPA5 being a functional LPA receptor on MCs, and suggests that LPA5 may be important for conveying signals leading to MIP-1β generation and release from MCs in contexts where LPA levels are present in the tissues as a result of injury, tumor invasion, or inflammation.
